# Medical malpractice in Norway: frequency and distribution of disciplinary actions for medical doctors 2011–2018

**DOI:** 10.1186/s12913-021-06334-2

**Published:** 2021-04-09

**Authors:** Martin B. Harbitz, Per Steinar Stensland, Birgit Abelsen

**Affiliations:** 1 Department of Community Medicine, UiT The Arctic University of Norway, Norwegian Centre for Rural Medicine, 9037 Tromsø, Norway; 2 Department of Global Public Health and Primary Care, University of Bergen, 5007 Bergen, Norway

**Keywords:** Patient safety, Rural practice, Primary care, Secondary care, Medical litigation system, Disciplinary action, Medical malpractice

## Abstract

**Background:**

Physicians who perform unsafe practices and harm patients may be disciplined. In Norway, there are five types of disciplinary action, ranging from a warning for the least serious examples of malpractice to loss of licence for the most serious ones. Disciplinary actions always involve medical malpractice. The aims of this study were to investigate the frequency and distribution of disciplinary actions by the Norwegian Board of Health Supervision for doctors in Norway and to uncover nation-wide patient safety issues.

**Methods:**

We retrospectively investigated all 953 disciplinary actions for doctors given by the Board between 2011 and 2018. We categorized these according to type of action, recipient’s profession, organizational factors and geographical location of the recipient. Frequencies, cross tables, rates and linear regression were used for statistical analysis.

**Results:**

Rural general practitioners received the most disciplinary actions of all doctors and had their licence revoked or restricted 2.1 times more frequently than urban general practitioners. General practitioners and private specialists received respectively 98.7 and 91.0 disciplinary actions per 1000 doctors. Senior consultants and junior doctors working in hospitals received respectively 17.0 and 6.4 disciplinary actions per 1000 doctors. Eight times more actions were received by primary care doctors than secondary care doctors. Doctors working in primary care were given a warning 10.6 times more often and had their licence revoked or restricted 4.6 times more often than those in secondary care.

**Conclusion:**

The distribution and frequency of disciplinary actions by the Norwegian Board of Health Supervision clearly varied according to type of health care facility. Private specialists and general practitioners, especially those working in rural clinics, received the most disciplinary actions. These results deserve attention from health policy-makers and warrant further studies to determine the factors that influence medical malpractice. Moreover, the supervisory authorities should assess whether their procedures for reacting to malpractice are efficient and adequate for all types of physicians working in Norway.

## Background

Patients affected by medical malpractice experience increased morbidity and mortality [1–3]. Unsafe medical practices, where patients are harmed by the medical care system designed to help them, are prevalent in both primary and specialized care [3–7] and the associated emotional and financial costs are substantial [1, 2, 8]. Medical litigation systems in different countries that address cases of malpractice by physicians vary in form [9–11]. However, the medico-legal principles are universal in that patients or their relatives must express a concern or file a complaint about a physician or a health institution. In order to be disciplined, the physician must, through his or her medical conduct, have provided substandard or negligent care that led, or could have led, to patient harm [9, 10, 12]. In Norway, this process is the responsibility of the National Board of Health Supervision (NBHS), which receives the most serious patient complaints and assesses whether a doctor should be disciplined. Unlike the litigation system in the United States, decisions by the NBHS do not award financial compensation to patients, and the NBHS assesses only the legal aspect, i.e. whether the health care provider is responsible according to health care legislation. In a few rare and extraordinary cases, civil courts also impose additional legal penalties.

Most recent studies aiming to determine the causes that led to disciplinary actions analysed types of medical error [9, 13] and characteristics of physicians (sex, age, profession and work experience) in relation to such actions [13, 14]. Based on the perspectives of Reason [15] and Donabedian [16] regarding quality and errors in health care, factors such as system design, organizational culture and lack of management or training can create ‘latent’ upstream errors that in the end cause ‘active’ patient harm. The factors involved can be external factors that are not under the control of a medical institution (e.g. geographical, political or cultural issues) or organizational factors (structure, organizational culture, working conditions) [17]. To discipline only individual doctors for mistakes created by these factors is not logical because errors are bound to continue until the underlying conditions are remedied. There are some indications that suggest that doctors who work in general practice receive more complaints than those who work in hospitals [18]. A Danish retrospective register study did not establish any relationship between general practitioner (GP) location (urban or rural) and the occurrence of malpractice complaints [19]. An Australian cohort study found a higher risk for complaints in remote areas in Australia than in urban areas [20]. The scarce evidence on the influence of external and organizational factors on medical malpractice warrants greater attention because a thorough evaluation might reveal important implications for improving patient safety. Therefore, the aim of this study was to investigate these factors and descriptive data of all doctors in Norway disciplined between 2011 and 2018.

## Methods

In this retrospective descriptive study, we analysed the frequency, trends, total and geographical distribution, rates and organizational factors of all doctors in Norway who were disciplined between 2011 and 2018. The dataset consisted of all disciplinary actions given to doctors in Norway. When dealing with whole population datasets, observed differences are considered de facto differences.

### The medical litigation system in Norway

**Fig. 1:**
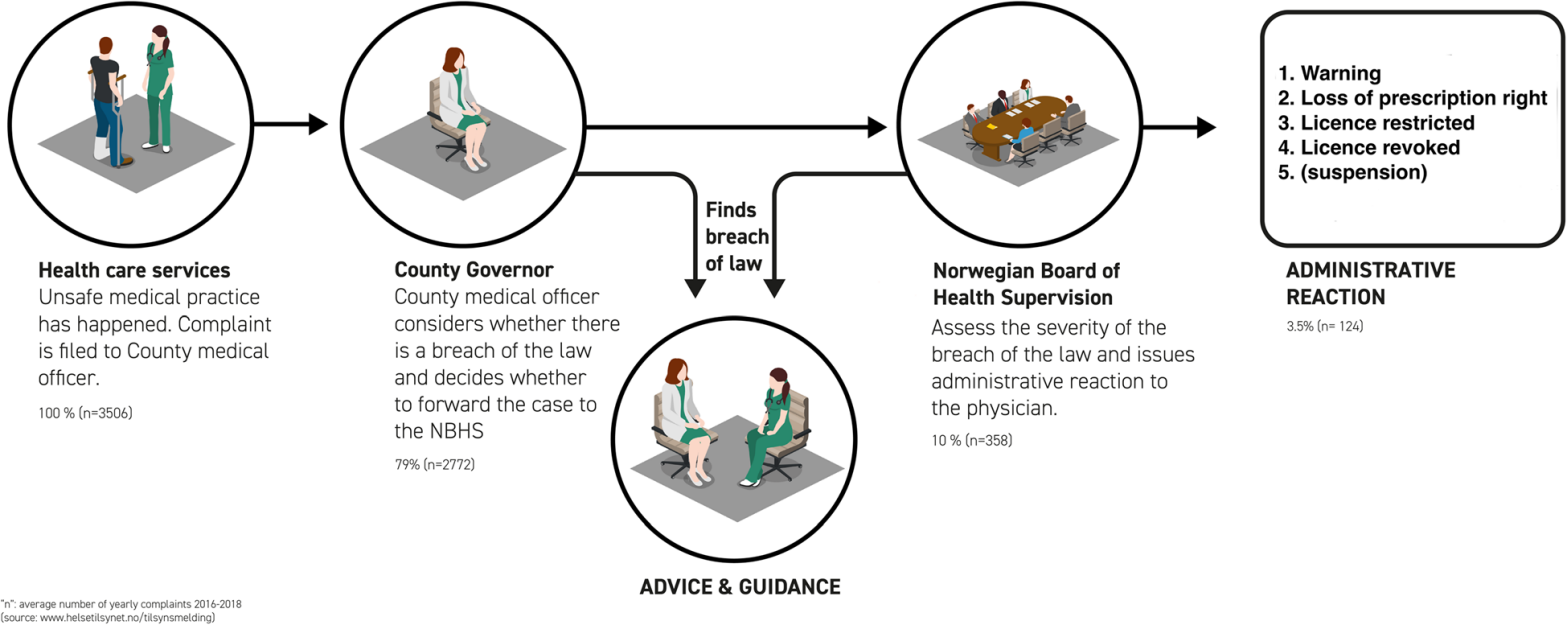
From medical malpractice to disciplinary action

In Norway, several acts regulate how patients, family members, health care personnel and health authorities can or must report medical malpractice to the NBHS. The event must have resulted in death or an unexpected serious outcome. Reported cases are usually first assessed by the local NBHS representative, the chief county medical officer. If the reported violation is serious and potentially irresponsible [12], the case is forwarded to the NBHS [21]. Figure 1 presents a flowchart of the reporting process.

There are five potential disciplinary actions; a warning is the least serious and having one’s licence revoked or suspended is the strongest form of action [12], see Fig. 1. The most frequent and serious patterns of violations by physicians include sexual misconduct, failure to meet the required standard of care and unprofessional conduct [9, 12, 13].

### The Norwegian health care system

In Norway, all inhabitants can choose their own local regular GP [22]. More than 4700 doctors are currently working as regular GPs [23], and are part of a patient list system which enables an enduring patient-GP relationship [24]. GPs are usually self-employed [25] and often share a local clinic with a few colleagues. Over half of Norway’s GP clinics are classified as rural [24]. For many of those living in rural areas, it takes more than 40 min to reach a local emergency primary care clinic [26], and substantially longer to reach a hospital. Physicians working in hospitals are employed by regional health authorities (state enterprises). There are four regional health authorities, which are responsible for 39 hospitals and over 12,900 physicians [27] (2018 figure). Hospitals provide the public with free specialized treatment. There are university hospitals, regional hospitals and smaller local hospitals that serve the inhabitants of a local area. In addition, there are almost 1000 private specialists working in Norway. They work in their own private facilities and provide specialist outpatient diagnostics that are almost equivalent to hospital diagnostics. The private specialists receive subsidies from the regional health authorities but are self-employed [28].

### Sample

The sample consisted of all disciplinary actions given to doctors in Norway between 1 January 2011 and 31 December 2018. After submitting a formal application to the Norwegian Centre for Research Data (project #53124) and a formal request to the NBHS, we were allowed access to a dataset for analysis. Data from the NBHS have been proven to be reliable and predictable [29]. The first author MBH examined each disciplinary action and corresponded with the NBHS if data were missing. MBH anonymized the dataset by replacing names of clinics and hospitals with a centrality index number [24] based on the geographic location. Every municipality in Norway has a centrality index number from 1 (most central) to 6 (least central). In the present study, we merged two consecutive index numbers (see Results) to represent urban areas (centrality index 1–2), semi-urban areas (centrality index 3–4) and rural areas (centrality index 5–6). The complete dataset contained 13 variables including registration date, issue date of the disciplinary action, type of action, speciality of the recipient physician and workplace centrality index number at the time of the medical error. The cause of the disciplinary action was not available due to privacy regulations. There were ten different types of physicians included in the dataset: 1) GPs in general practice, 2) GPs in emergency primary care clinics, 3) nursing home doctors, 4) private specialists, 5) medical interns, 6) junior hospital doctors, 7) senior hospital consultants, 8) company doctors, 9) other doctors and 10) licensed medical students.

### Data analysis

Descriptive statistics were used to analyse the dataset. MBH performed frequency counts and cross-tabulation of variables to calculate annual frequency and distribution of disciplinary actions. We specified linear regression models (Y = a + bX) to analyse for significant trends in actions over time (Y_i_ = actions in year i, X_i_ = year i, i = 1,…8). Rates of disciplinary actions were calculated per 1000 physicians. Comparative rate analysis was performed by basic division. Statistics Norway, the Norwegian Medical Association and the Norwegian Directorate of Health provided activity data and information on services. Geographical distribution of doctors was only available for GPs. Because of privacy considerations, cases involving suspension and revocation of specialization licences (*n* = 4) were not further analysed. Furthermore, doctors disciplined outside Norway (*n* = 110) were not analysed. We considered GPs in general practice, GPs working in emergency primary care clinics and nursing home doctors as representing primary care doctors, while junior hospital doctors and senior hospital consultants were grouped as secondary care doctors. The data were analysed using IBM SPSS Statistics 26 (IBM Corp. Statistics 26, SPSS Inc. 2019, USA).

## Results

The NBHS provided a dataset of 953 disciplinary actions. Three of these (0.4%) lacked geographic location and were thus excluded from the analysis.

### Annual frequency

Table 1 shows the annual and total frequencies of disciplinary actions for physicians by the NBHS in the study period. A total of 950 disciplinary actions were taken, and 57% of these were warnings, while 36% involved the revocation or restriction of a licence.

**Table 1:** Disciplinary actions. Total and by reaction type. 2011–2018. Frequency and percent

	2011 n (%)	2012 n (%)	2013 n (%)	2014 n (%)	2015 n (%)	2016 n (%)	2017 n (%)	2018 n (%)	Total n (%)
**Disciplinary action type**									
Warning	59 (61)	71 (59)	64 (62)	54 (55)	99 (62)	62 (57)	58 (47)	78 (56)	545 (57)
Licence restricted	6 (6)	7 (6)	7 (7)	6 (6)	14 (9)	10 (9)	12 (10)	10 (7)	72 (8)
Loss of prescription right	7 (7)	12 (10)	7 (7)	6 (6)	8 (5)	7 (6)	7 (6)	7 (5)	61 (6)
Licence revoked	23 (24)	30 (25)	25 (24)	32 (33)	38 (24)	28 (26)	47 (38)	45 (32)	269 (28)
Suspension/loss of specialization approval	2 (2)	0 (0)	0 (0)	0 (0)	1 (1)	1 (1)	0 (0)	0 (0)	4 (0)
**Total**	97 (100)	120 (100)	103 (100)	98 (100)	160 (100)	108 (100)	124 (100)	140 (100)	950 (100)

### Trends

**Fig. 2:**
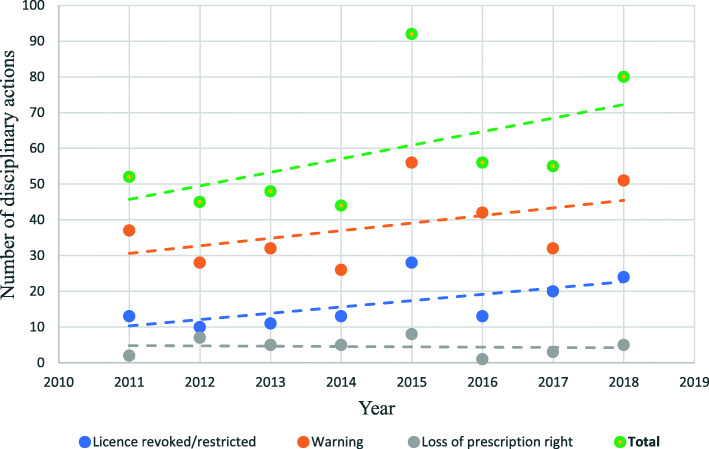
Trends in disciplinary actions over time for GPs in Norway (2011–2018)

**Fig. 3:**
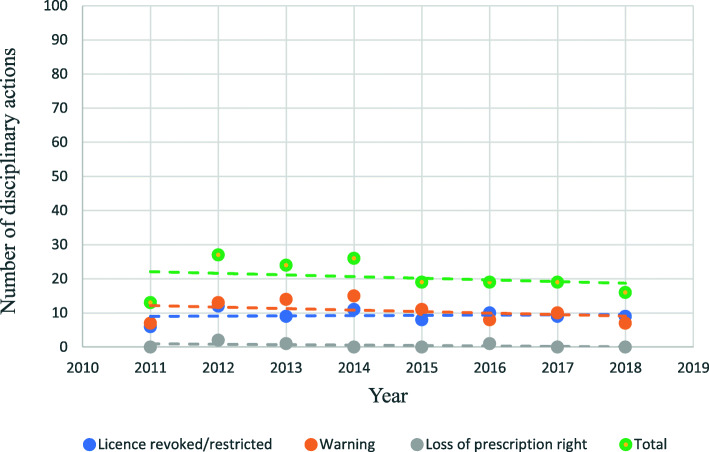
Trends in disciplinary actions over time for secondary care doctors in Norway (2011–2018)

For GPs (Fig. 2) none of the linear regression models showed statistically significant time trends: total number of disciplinary actions (b = 2.58, *p* = .41), warnings (b = 1.07, *p* = .58), loss of prescription rights (b = −.18, *p* = .65), and revocation/restriction of licence (b = 1.7, *p* = .12). A similar analysis for secondary care doctors (Fig. 3) also revealed no significant time trends; total number of disciplinary actions (b = − 0,49, *p* = .57), warnings (b = −.50, *p* = .36), loss of prescription rights (b = −.12, *p* = .68), and revocation/restriction of licence (b = .13, *p* = .68).

### Total and geographical distribution

Table 2 presents the distribution of disciplinary actions given between 2011 and 2018 to different types of doctors. The presentation only includes disciplinary actions originating from Norway. Primary care physicians were given 70% of all warnings, 79% of all losses of prescription rights and 57% of revocations/restrictions of licences. For secondary care physicians, the respective numbers were 16, 9 and 27%. Other categories of doctors accounted for 14, 11 and 16%, respectively.

**Table 2:** Disciplinary actions (in Norway) by type of action and doctor, 2011–2018

	Warning n (%)	Loss of prescription rights n (%)	Licence revoked or restricted n (%)	Suspension/revocation of specialization licence n (%)	Total n
**Primary care doctors**					
GPs in general practice	307 (57)	33 (77)	126 (49)	1 (25)	467
GPs in emergency clinics	58 (11)	0 (0)	16 (6)	0 (0)	74
Nursing home doctors	9 (2)	1 (2)	4 (2)	0 (0)	14
**Secondary care doctors**					
Junior doctors	16 (3)	1 (2)	19 (7)	0 (0)	36
Senior consultants	70 (13)	3 (7)	51 (20)	1 (25)	125
**Other types of doctors**					
Private specialists	59 (11)	4 (9)	26 (10)	0 (0)	89
Medical interns	5 (1)	1 (2)	2 (1)	0 (0)	8
Company doctors	0 (0)	0	0 (0)	1 (25)	1
Other doctors	11 (2)	0	13 (5)	1 (25)	25
Licensed medical students	0 (0)	0 (0)	1 (0)	0 (0)	1
**Total n (%)**	**535** (100)	**43** (100)	**258** (100)	**4** (100)	**840**^a^

^a^110 disciplinary actions occurred outside Norway

Table 3 presents the total number of disciplinary actions by centrality of the GP’s workplace municipality. Table 4 shows the rates of actions for the same GPs. Rural GPs received 1.7 times more disciplinary actions than their urban colleagues (148.9/88.4). For the most serious type of action, rural GPs had their licence revoked or restricted 2.1 times more frequently than GPs in urban areas (47.4/22.5). Regarding the type of disciplinary action linked to unprofessional handling of medication, urban GPs had 2.1 times more cases of loss of prescription rights than rural GPs (8.4/4.1).

**Table 3:** Total numbers of disciplinary actions for GPs by type of action and centrality of workplace municipality, 2011–2018

^a^	Warning	Loss of prescription rights	Licence revoked or restricted	Total
Urban GPs	110	16	43	**169**
Semi-urban GPs	125	14	48	**187**
Rural GPs	72	3	35	**111**
**Total**	307	33	126	**467**

^a^Average number of GPs (2016–2018) split by centrality index https://www.ssb.no/statbank/table/12720/tableViewLayout1/

**Table 4:** Rates of disciplinary actions per 1000 GPs by type of action and centrality of the workplace municipality, 2011–2018

^a^	Warning	Loss of prescription rights	Licence revoked or restricted	Total	No. of consultations per action^b^	Number of municipalities^c^
Urban GPs	57.5	8.4	22.5	**88.4**	2.9 ∗ 10^5^	68
Semi-urban GPs	60.3	6.8	23.2	**90.3**	2.6 ∗ 10^5^	190
Rural GPs	97.4	4.1	47.4	**148.9**	1.2 ∗ 10^5^	170

^a^Average number of GPs (2016–2018) split by centrality index https://www.s00sb.no/statbank/table/12720/tableViewLayout1/

^b^Total number of consultations (2011–2018) by GPs in each workplace municipality. Data provided on request by Statistics Norway

^c^Report “New Centrality Index for Municipalities”, Statistics Norway, 2017, ISBN 978-82-537-9627-7, Oslo

### Rates and organizational factors

Table 5 presents the rates of disciplinary actions per 1000 doctors among different categories of doctors. GPs received the most actions at 98.7 per 1000. A warning was given 2.4 times more frequently than revocation or restriction of the licence. GPs in general practice received 1.6 times more disciplinary actions than GPs in emergency primary care clinics and 3.7 times more disciplinary actions than nursing home doctors. Private specialists received the second highest proportion of disciplinary actions, and the most actions per consultation. Private specialists were also the group with the highest proportion of doctors with a specialization degree.

**Table 5:** Rates of disciplinary actions per 1000 doctors, and actions per 1 million consultations, by type of action and doctor. 2011–2018

	Disciplinary actions per 1000 doctors				Consultations		Demographics	
	Warning	Loss of prescription rights	Licence revoked or restricted	Total	Consultations per year^a-c^	Actions per 1 million consultations	Number of doctors^d-i^	Percentage of specialists
**Primary care doctors**					15,681,763	4.42		49.1
GPs in general practice	65.0	7.0	26.7	**98.7**			4724	
GPs in emergency clinics	49.3	0.0	13.6	**62.9**			1175	
Nursing home doctors	16.9	1.9	7.5	**26.3**			533	
**Secondary care doctors**					11,026,425	1.82		
Junior doctors	2.8	0.2	3.4	**6.4**			5601	10.9
Senior consultants	9.6	0.4	7.0	**17.0**			7312	94.6
**Other types of doctors**								
Private specialists	60.3	4.1	26.6	**91.0**	2,006,196	6.04	978	99.6
Medical interns	6.2	1.2	2.4	**9.8**			817	

^a^Average number (2016–2018) of consultations by GPs in general practice, GPs in emergency clinics and nursing home doctors (estimated using 4 contacts/bed/year) https://www.ssb.no/helse/statistikker/fastlegetj & https://www.ssb.no/statbank/table/10903/ & https://www.ssb.no/pleie/

^b^Average number (2016–2018) of contacts in somatic and psychiatric hospitals. https://statistikk.helsedirektoratet.no/bi/Dashboard/37f4e0dd-61fd-4846-a7c1-d87553ce2c1a?e=false&vo=viewonly & https://www.helsedirektoratet.no/rapporter/aktivitetsdata-for-psykisk-helsevern-for-voksne-og-tverrfaglig-spesialisert-rusbehandling

^c^Average number (2016–2018) of contacts for private specialists. https://www.helsedirektoratet.no/rapporter/aktivitetsdata-for-avtalespesialister

^d^Average number of GP contacts between 2016 and 2018. Source: https://www.ssb.no/statbank/table/12720/

^e^Full-time equivalent GPs in emergency primary care centres. Report from National Register 2018, National Centre for Emergency Primary Health Care, Tone Morken, Norway

^f^Average number of physicians employed in nursing homes and institutions in 2011 and 2013–2017. From the report “Physicians in primary and secondary care”, 2018, IS-2789, OSLO: Norwegian Directorate of Health

^g^Average number of licensed specialists between 2011 and 2018. Source: https://www.ssb.no/statbank/table/03750

^h^Average number of working physicians < 70 years, 2011–2018. Source: physician statistics, Norwegian Medical Association: https://legeforeningen.no/Emner/Andre-emner/Legestatistikk/Yrkesaktive-leger-i-Norge/Stillingsgrupper/

^i^Average number of medical interns employed between 2013 and 2017. From the report “Physicians in primary and secondary care”, 2018, IS-2789, OSLO: Norwegian Directorate of Health

In hospitals, senior consultants received the most disciplinary actions. Per 1000 senior consultants, 17.0 received a disciplinary action. Junior doctors received 6.4 disciplinary actions per 1000 doctors, 2.7 times fewer than senior consultants.

The rate comparisons between primary and secondary care doctors revealed that primary care doctors received 8.0 times more disciplinary actions than secondary care doctors. Further, primary care doctors received a warning 10.6 times more often, had their licence revoked or restricted 4.6 times more often and lost their prescription rights 14.8 times more often than secondary care doctors. Rural GPs, the group with most disciplinary actions per 1000 physicians (148.9), received such actions 8.7 times more frequently than senior consultants and 23.3 times more frequently than junior doctors.

## Discussion

In this study, we investigated the distribution and frequency of disciplinary actions given to physicians in Norway between 2011 and 2018. Our findings reveal considerable differences. One of the core findings in this study is that rural GPs had the highest rate of disciplinary actions among all physicians. Furthermore, GPs and private specialists had higher rates than other groups of physicians. Because a disciplinary action is a sign of medical malpractice and a possible indicator of problems related to patient safety, we will discuss our findings in the context of the research aims.

### Organizational and systemic factors

According to our findings, physicians who work in small clinics or alone (GPs and private specialists) had respectively 4.3 and 3.9 times higher rates of disciplinary actions than those working in large organizations (hospital doctors). This difference may partly be explained by the supervisory system of the NBHS. Based on its system-wide perspective of patient safety [6, 15, 16, 30–32], the NBHS seeks to identify systemic causes as a primary goal when a medical error occurs [33]. The theory is that addressing a single systemic error will be more efficient in benefitting more future patients than reacting to a single medical error, thus enhancing health care services for the future. Many individuals are involved in health care in hospitals, while in general practice and private specialist clinics, much of the organization is de facto the physician. For example, an acutely ill patient arriving at hospital would interact with a large group of health care workers before diagnosis and treatment were initiated. If the same patient came to a GP clinic or a private specialist, he or she would interact with a health care secretary and one GP or one private specialist. Thus, clinical decisions and patient responsibility clearly vary between these two contexts. Despite providing very different types of health care, GPs and private specialists have almost identical frequencies of disciplinary actions. The apparent focus of the NBHS on system causality and the lack of system protection in primary care make GPs and private specialists more vulnerable to disciplinary actions.

### More disciplinary actions for rural GPs

Comparing the GPs in our study, we found that rural GPs received 1.7 times more disciplinary actions than their urban counterparts. A Danish study reported no statistically significant association between litigation figures and rurality [19], although Australian researchers found more patient complaints [20] in this context. Rural GP clinics face challenges in care provision in terms of accessibility, limited health care services, use of locums and issues related to vast distances and transportation [34, 35]. Rural patients have been found to report lower levels of relational continuity [35]. In a recent interview-based study on rural general practice patients, we found that patients were more willing to accept mistakes and errors by their regular GP than by locum GPs [36]. If we assume that continuity of care is an important quality indicator of health care [32], one hypothesis is that the use of locums or GPs on short-term contracts [37] results in more cases of medical malpractice in rural areas. Another possible explanation is that being located far from hospitals may affect rural GPs’ clinical decisions [38]. Furthermore, rural GPs see, almost exclusively, all the acutely ill patients, whereas in urban areas, more specialists are available, and these patients can bypass GPs by being taken by ambulance directly to hospital. Many rural GPs work frequent shifts in emergency primary care units, possibly resulting in fatigue, sleep deprivation and cognitive overload, all of which are risk factors for committing errors [39].

### Strengths and limitations

Using the NBHS national database for analysis, all disciplinary actions were processed equally and uniformly at a national centre, avoiding different types of selection or affective bias. The datasets from the NBHS and Statistics Norway were complete and trustworthy, providing the opportunity for a nationwide analysis and new knowledge.

The 950 disciplinary actions must, however, be interpreted in the context of approximately 230 million patient contacts that occurred between 2011 and 2018. Our findings do not represent all occurrences of medical malpractice in Norway. There is a possibility of selection bias, as some serious complaints could have been addressed by the local chief county medical officer instead of being forwarded to the NBHS [40]. In the present study, we addressed some external and system factors affecting disciplinary actions, keeping in mind a famous quote of Donabedian: ‘Systems…are enabling mechanisms only. It is the ethical dimension of individuals that is essential to a system’s success’.

### Implications for practice

Our findings demonstrate the potentially vulnerable position of doctors working alone and in small clinics. The organizational and systemic factors designed to support doctors may be weak in some of the small rural clinics where the disciplinary actions were the most frequent. There seems to be an unexploited potential to improve patient safety by offering doctors in these clinics a stronger support system. There was a marked difference between primary and secondary care doctors in the rates of disciplinary actions given, indicating a higher degree of system protection in secondary care facilities.

Future research should analyse other types of data, and include case studies and in-depth qualitative studies to investigate why GPs, especially rural GPs, are more frequently disciplined.

## Conclusions

There are clear differences in the distribution and frequency of disciplinary actions given by the NBHS to physicians working in different health care settings. Private specialists and GPs, especially those working in rural clinics, received the most disciplinary actions. These results warrant the attention of health care leaders responsible for ensuring patient safety. Hopefully they will be inspired to initiate further studies to identify the main factors influencing medical malpractice. The results of this study may also assist supervisory authorities in their quality assessments to determine whether their disciplinary system is efficient and adequate for all the different categories of physicians working in health care in Norway.

## Data Availability

A de-identified dataset might be made available upon reasonable request of the authors. This will need approval by the NBHS and NSD to ensure anonymity and data protection.

## Abbreviations

GP: General practitioner
NBHS: The Norwegian Board of Health Supervision
